# Medical Gossip

**Published:** 1861-07

**Authors:** 


### Art. XII.—MEDICAL GOSSIP.

The little "difficulty" between the Apothecaries' Society and the College of Physicians has at length been brought to the arbitrement of the law. In the course of the past quarter the question of the right of the College of Physicians to form a new order of licentiates, who might practise pharmacy, was brought before Vice-Chancellor Sir W. P. Wood. On the part of the Society of Apothecaries it was urged, that the Medical Act of 1858 recognised and assigned to each branch of the profession its distinct powers and sphere of action, and that it was a fraud upon that statute for the College of Physicians to create, in violation of their own by-laws, a new body of practitioners altogether unknown in the profession, and, so to speak, of an "epicene" order, partly physicians, partly apothecaries. The rights of the apothecaries, it was said, which had been secured to them by the Act of 1815, would be seriously invaded if the College were permitted to carry out its design. Moreover, it was important upon grounds of general policy that the College should be restrained from so doing, lest the public should be flooded with unqualified men, whose ignorance in the proper dispensing of medicines might occasion serious harm. It was essential therefore to the public welfare, the ancient dignity of the College, the authority and privileges of apothecaries, and the maintenance in their integrity of the provisions of the Legislature, that the Court should interpose to prevent this unauthorized attempt by the College of Physicians to do that which was not only *ultra vires*, but would go far to destroy the rank and position enjoyed by the College for more than three centuries.

Notwithstanding this pathetic appeal, in which the Society of Apothecaries manifested not merely a due care for its own welfare, but also a greater care for the dignity of the College of Physicians than that College had thought fit to show for itself, the Court manifested a complete insensibility to the considerations urged upon it by the appellants. The Vice-Chancellor held that their arguments were of no weight, and that in respect of the matter complained of, the College of Physicians must be left to its own devices.

He said, that in order to support the argument that the creation of a new order of licentiates by the College was a fraud upon the Medical Act of 1858, it would be necessary to assume that the *status* of licentiate was definitely fixed, and must now, after the passing of that Act, be taken as stereotyped and bound for ever by the former conditions and restrictions. But what were the conditions imposed by the by-laws of the College? They might be divided into two classes—conditions precedent, as to the capacity and education, &c. of the candidates; and conditions subsequent, as to the compliance with the by-laws (restricting the vending of drugs, &c.) after receiving the licence. In one sense this prohibition was a condition precedent, but it would be more convenient to treat it as a condition subsequent. What evidence, then, he asked, was there that these conditions were to remain unalterably fixed, as contended for by the Apothecaries' Society? On the face of the Medical Act there was nothing to that effect—nothing to restrict the by-laws to their existing state. How could it be contended that the College of Physicians were not to have full power to modify, from time to time, the qualifications required for admission into their body, with the control of the general council? The only point on which the Legislature interfered in this respect was that particular theories—such as homœopathy, mesmerism, and the like—were not to act as disqualifications. Even assuming that the College was precluded by the Act from relaxing its conditions subsequent, he was by no means sure that this would be sufficient to sustain the bill, or that the licences granted by the College must not be left to be tested at their worth in a court of law. The real question was how far the design of the defendants was a fraud upon the Legislature, and how far they were precluded from altering their own by-laws. Their original charter admitted the whole domain of physic within its purview. Not only "physic," but surgery was included, and its effect was to throw the universal medical science under the control of the College of Physicians. Up to the 15th of James I. no one could have disputed the right of the College to deal with the question of selling medicines, and to revoke any by-laws made by them upon the subject. It might be observed that the very existence of such a by-law implied that it would have been the custom of physicians to deal in drugs but for such a restriction. No doubt, considerable inconvenience was occasioned to the public from the restriction, and this gave rise to the apothecaries and their incorporation by James I. The apothecaries, from the very humble position of grocers and mere drug-sellers, gradually acquired greater knowledge as to the properties of drugs, and skill in their administration. They were accordingly incorporated by charter from James I., with certain privileges to themselves and restrictions against others. It could not be maintained for an instant that this charter had in any way interfered with the privileges of the College of Physicians granted by charter and confirmed by statute. From the time of Henry VIII. down to 1815 it was not possible to suggest a doubt that the College might have relaxed any of their by-laws, including that which restricted the sale of drugs. Then came the Apothecaries' Act of 1815. It was contended that the saving clause (sec. 29) did not preserve to the College the right, which existed before the Act, of granting licences to practise physic divested of the restriction contained in their by-laws. To say the least, the matter was far too doubtful to induce the Court to restrain a public body acting *bonâ fide* and in the belief that they were not exceeding their functions, nor was it clear that that Court was the medium through which a solution of the question could be properly obtained. The Vice-Chancellor then adverted to the case of surgeons dispensing medicines and recovering for them. There was no express repeal of the privileges of the College of Physicians, but on the contrary, an express saving, and unless the two things were so absolutely inconsistent that one of them must of necessity be excluded, he could not come to the conclusion that the privileges vested in the College were abrogated by section 20 of the Apothecaries' Act (imposing a penalty of 20*l.* upon any one acting or practising as an apothecary without the licence of the Society). The Vice-Chancellor, after some further observations, proceeded to consider the Medical Act of 1858. It had been contended that as the Legislature must have been aware of the previous *status* of licentiates, the creation of a new body of licentiates would be a fraud upon the Act. But until 1858 it was clear that the College had full power to create that new body, and if it were necessary to determine the question upon this ground, he thought that the Act of that year did not abrogate such right. There was not a *scintilla* of evidence on the face of the Act of an intention to deprive any one of the medical bodies of their power to alter their by-laws, so long as they kept within the limits of their charter. If he were to import into the case the knowledge possessed by the Legislature, all that could be assumed would be a knowledge of the exact state of things then existing; and Parliament must be taken to have known that by-laws could always be altered by the bodies who had made them. The Vice-Chancellor then referred to the preamble in support of his view, that there was nothing in the Act to abrogate the powers existing in the several medical bodies. The only grievance alleged in the information was that the defendants were elevating the old licentiates into members of the foundation, as it were, and creating a new body inferior in qualifications to the former. The question, however, returned to this :—Had the defendants authority to do this act? The information did not state that this new body would be apothecaries, but "to some extent a class of apothecaries." In his view of the case no possible amendment would help the information. Assuming, for the moment, that the plaintiffs would have a right of action against these new licentiates, who took their licences with knowledge of the Act of 1815, still the College of Physicians would not degrade their licentiates for selling drugs, and the worst that would happen would be that they might have to obtain the certificate from the Society of Apothecaries. As to the argument of public injury arising from the harm done to these young men who accepted the licences from the College, and were consequently deceived, he confessed that he did not attribute much weight to it. The legal maxim, "*Volenti non fit injuria*," would apply to such a case. In conclusion there was not a shadow of a pretence for saying that down to 1815 the privileges originally conferred upon the College of Physicians had been in any way abrogated. The Act of 1815 did not, in his opinion, prevent the College from altering their by-laws, and clearly the Act of 1858 had not that effect, so long as they kept within the limits of their charter. Upon these grounds the case of the Society of Apothecaries must fall to the ground.

Whether the apothecaries will try their case at common law remains to be seen, but the lot that has thus far befallen them in the present conjuncture ought, we would suggest, to have a moral for the College of Surgeons. What if that worthy corporation should not amend such of its ways as are open to reprehension, the College of Physicians should step in and claim its right of making surgeons? The statute 32 Henry VIII. is sufficiently precise upon this point. "And forasmuch," we read, "as the science of Physick doth comprehend, include, and contain the knowledge of Surgery, as a special member of the same, Therefore be it enacted, That any of the said Company or Fellowship of Physicians, being able, chosen, and admitted by the said President and Fellowship of Physicians, may from time to time, as well within the City of *London*, as elsewhere within the Realm, practise and exercise the said science of Physick in all and every his members and parts; any Act, Statute, or Provisions made to the contrary notwithstanding."

In May, 1860, the Council of the Royal Medical and Chirurgical Society appointed a committee "for the purpose of conferring with some of the Medical Societies of London, on the subject of a proposition for uniting in one comprehensive body the various Societies engaged in the prosecution of separate departments of Medical Science." The Presidents of the Epidemiological, Pathological, and Obstetrical Societies were communicated with, and the councils of those societies immediately appointed committees to take the subject mooted into consideration, in conjunction with the committee appointed by the Medico-Chirurgical Society.

The last-named society was represented by Mr. Skey, the president; Dr. Williams, and Mr. Spencer Smith, vice-presidents; Mr. Alexander Shaw, the treasurer; Mr. Hawkins, and the secretaries, Dr. Barclay and Mr. C. H. Moore. The Epidemiological, by Dr. Babington, the president; Dr. Milroy, Mr. J. F. Marson, and the secretary, Dr. MacWilliam. The Pathological, by Mr. Fergusson, the president; Dr. Murchison, and the secretaries, Dr. Ogle and Mr. Henry Thompson. The Obstetrical, by Dr. Rigby, the president; Dr. Tyler Smith, and the secretaries, Dr. Graily Hewitt and Dr. Tanner. The united committee, at its first meeting, resolved unanimously—

"That it is the opinion of this Meeting that it would tend to the advancement of Medical Science were the Royal Medical and Chirurgical, the Pathological, the Epidemiological, and the Obstetrical Societies united under one head, and these different branches of Medical Science carried out in corresponding sections of one Society."

Subsequently the following scheme was also agreed to by the United Committee:—

I.—That the united Society be divided into the following Sections:

1. Practical Medicine and Surgery.
2. Pathology and Morbid Anatomy.
3. Epidemiology and Hygienics.
4. Obstetrics and Diseases of Women and Children.
5. Physiology (including Anatomy and Animal Chemistry).
6. Psychological Medicine.
7. Medical Jurisprudence.

II.—That the Treasurers of each Section respectively receive the subscriptions to such Section, and defray from their own funds the expense of publishing their Transactions, and other necessary outlay. That the surplus, if any, be paid into the General Fund, and any deficiency be supplied from that fund.

III.—That Fellows of the Royal Medical and Chirurgical Society [*i.e.* of the Society when combined] be Members of all the Sections, and have a right to attend all Meetings of such Sections.

IV.—That persons, not Fellows of the Society, be admitted Members of any particular Section on payment of an annual sum, and be designated Members of such Section, and Associates of the Royal Medical and Chirurgical Society [*i.e.* the Societies when combined].

V.—That each Section elect annually a President and other officers for the management of the affairs of its own department, and also from time to time elect Members who are not Fellows of the Society.

VI.—That in the annual nomination of Fellows recommended by the Council for election as President and Council of the united Society for the ensuing year, two at the least be selected from among the Members of Committee of Management of each of the several Sections.

VII.—That Members of particular Sections have the right to attend all meetings of such Section, and to be admitted to the use of the Reading-room, but not to remove from the Library any books, except such as belong to the Section.

VIII.—That it be the business of the Committee of each Section to prepare a report of the proceedings of the past Session, to be read at an Annual Meeting to be held for that purpose.

On the 5th April, a special meeting of the Medico-Chirurgical Society, by a majority of 38 against 23, affirmed the resolution, that the amalgamation of the different societies would prove advantageous to medical science; and subsequently the Epidemiological Society came to the same conclusion. The Obstetrical and Pathological Societies, it is understood, dissent from the resolution.

The promoters of the scheme for amalgamation appear to hold that the union would not only give an impetus to and promote in several ways the advancement of medical science, but also that the combined society would worthily represent English scientific medicine, as well with the public as the profession, and both at home and abroad—opinions in which we heartily concur. The recalcitrancy of the two last-named Societies must, we fear, prove fatal to the scheme.

It has usually been the practice when an active dissidence of opinion has taken place between any two practitioners in consultation upon a case, whether the dissidence arose upon a question of diagnosis or of treatment, for one to retire from the case. The practice was consistent with common sense; and indeed, the very supposition of a continuation of consultations under such circumstances, professedly for the welfare of the patient, involves an absurdity of the most glaring description. It never occurred to orthodox medical practitioners that, in adopting this line of conduct, they were necessarily guilty of disrespect to patients or friends; and, on the other hand, it seemed pretty apparent that to adopt an opposite course would be to countenance a somewhat discreditable sham. We are now to learn that this rule of conduct, although very fit and proper as it applies to orthodox practitioners of medicine, by no means holds good of the relations between orthodox and heterodox practitioners.

> "Oaths are not purposed more than law
> To keep the good and just in awe,
> But to confine the bad and sinful,
> Like mortal cattle in a pinfold."

Thus a medical man, of high local standing, has recently not only held a series of consultations with an homœopathic practitioner, but, having fallen in consequence under the ban of his professional neighbours, he has sought to justify publicly his conduct, and even affects to be astonished at their susceptibility. We keep back, as altogether too transcendental in this case, the reasons which might be supposed to influence a member of an honourable profession, when brought into contact with a professor of perhaps the most stupendously impudent form of quackery that the world has yet seen.

We will grace the homœopath so far as to apply to him, for the nonce, a rule of conduct observed by regular practitioners of medicine. But even this leads us into an awkward dilemma; for so diametrically opposed are the creeds, pathological and therapeutical, of the regular practitioner in medicine and the homœopath, that no consultation between them is possible except by a sacrifice of principles on the part of one or the other, or of both. On the simple grounds of dissidence of opinion alone, therefore, consultation with a homœopath does not admit of justification.

The *Times* of June 4th contained the following telegraphic message:—

"*June 3rd.* On Sunday Count Cavour passed a restless night. He was bled this evening for the sixth time, and is now better. His physicians declare his illness to be a very mild form of typhus fever, without any very alarming symtoms."

The first impression on reading this extraordinary despatch was, that it was a sorry but cruel jest. In the last number of this Journal * we printed a curious letter from the English Ambassador in Spain to Queen Elizabeth, illustrative of the practice of an Italian physician in the sixteenth century. He was in attendance upon the Queen of Spain in a febrile attack, and literally bled her until he could obtain no more blood—for after repeated bleedings from the arm, "searching to let more blood at her nose and other places (not one drop would follow, no more than of twenty ventoses or more applied all at once to sundry parts of her body)." Her Most Catholic Majesty was in fact bled to death's door, and escaped by miracle. It seemed to us inconceivable that an Italian physician of any reputation in the nineteenth century would use the lancet as freely as it had been used in the sixteenth, and hope that at the worst the statement of the treatment to which Count Cavour had been subjected would prove to be a covert satire on Italian medical practice. "Mon père, monsieur, est toujours malade de plus en plus," says the countrywoman to the physician in *Monsieur de Pourceaugnac.*

---

* See Art. *Miscellanea Medica.*

" Ce n'est pas ma faute," he replies ; "je lui donne des remèdes : que ne guerit-il ? Combien a-t-il été saigné de fois ?" " Quinze, monsieur, depuis vingt jours." " Quinze fois saigné ?" " Oui." "Et il ne guérit point?" " Non, monsieur."*

But alas ! on June the 6th Count Cavour, the great hope of Italy died, and on the same day the *Times* correspondent wrote :—

" *Turin, June 6th.*

" *Consummatum est.* Count Cavour died this morning at seven o'clock. It was understood yesterday that the night would be critical, and the issue of the crisis was scarcely doubtful, as the sufferer had not rallied in the morning, as had been the case with him in the previous days. All power of reaction had left him, and the new attack of fever was sure to find him powerless.

" The Romans, it is said, crowned on the Capitol the physician who rid them of Pope Adrian VI. The Italians of our own days would honestly hang Count Cavour's doctors if the execution would afford any relief to their feelings. There never was a clearer case of a man murdered by his medical attendants. Within a very short period of five days they attempted to cure the Count of four or more different complaints,—congestion of the brain, typhus fever, intermittent fever, brain fever, dropsy, and lastly, gout ; and for all these diseases they could think of nothing but their own sovereign remedy—the lancet. I think these excellent practitioners are worthy to send down their names to posterity. They were,—Dr. Rossi, Dr. Mattoni, and towards the end, the King's physician, Riberi, the same in whose hands the mother, wife, and brother of Victor Emmanuel expired, one by one, in the early months of the fatal year 1855. Dr. Tommasi, who was summoned from Pavia by Cavour's friends, was not admitted to consultation.

" Notwithstanding frequent fits of delirium, Count Cavour seemed to have a distinct presentiment of his fate. Seeing himself alone with his domestic attendants, yesterday, he asked with great serenity, 'whether his doctors had forsaken him ?' On being answered with surprise and concern, that they could never have thought of leaving him for a moment, he replied with a smile, '*Domattina, gli abbandonero io*' ('It is I who shall quit them to-morrow morning') !"

Let us hope for the credit of medicine, that popular report has played false with the details of both diagnosis and treatment.

---

* Act i. sc. 7.

